# Association Between Sedentary Behavior and Primary Dysmenorrhea in Young Korean Women: A Cross-Sectional Online Survey

**DOI:** 10.3390/healthcare13101098

**Published:** 2025-05-08

**Authors:** Myongsook Hyun, Jaehee Kim

**Affiliations:** 1 Department of Alternative Medicine, Graduate School, Kyonggi University (Seoul Campus), 24, Kyonggidae-ro 9-gil, Seodaemun-gu, Seoul 03746, Republic of Korea; clavi21c@naver.com; 2 Graduate School of Alternative Medicine, Kyonggi University (Seoul Campus), 24, Kyonggidae-ro 9-gil, Seodaemun-gu, Seoul 03746, Republic of Korea

**Keywords:** sedentary behavior, dysmenorrhea, cross-sectional studies, young adult

## Abstract

Background/Objectives: Sedentary behavior is an independent risk factor for various health conditions, but its association with dysmenorrhea has been little investigated. This study aimed to examine whether sedentary behavior is independently associated with primary dysmenorrhea in young women, controlling for moderate-to-vigorous physical activity and other known risk factors. Methods: An online survey was conducted in 603 young women in South Korea in 2023. Menstrual pain intensity was measured using a numeric rating scale, and symptoms were assessed with the Cox Menstrual Symptom Scale. Sedentary behavior and physical activity were assessed using the Global Physical Activity Questionnaire. Known risk factors for dysmenorrhea included menstrual and lifestyle characteristics, sleep quality, and stress. Results: Longer sedentary time (hours/day) was correlated with more frequent (*r* = 0.144; *p* < 0.001) and severe (*r* = 0.123; *p* < 0.01) menstrual symptoms but not with pain intensity. Multiple linear regression analysis showed that sedentary time was independently associated with the frequency (*β* = 0.10; *p* < 0.01) and severity (*β* = 0.09; *p* < 0.05) of menstrual symptoms after adjusting for physical activity and other risk factors for dysmenorrhea. Multiple logistic regression analysis showed that women with higher levels of sedentary time had 1.05 times greater odds (95% CI, 1.00–1.10; *p* < 0.05) of experiencing severe pain compared to those with less sedentary time, even after adjusting for physical activity and other risk factors. Conclusions: Prolonged sedentary behavior in young women is associated with more frequent and severe menstrual symptoms, including more intense menstrual pain. These findings highlight the need for public health strategies that reduce sedentary behavior to alleviate dysmenorrhea.

## 1. Introduction

Primary dysmenorrhea refers to painful menstrual periods without an identifiable pathological cause and is a major global health issue among young women [1]. A meta-analysis study reported a global prevalence of dysmenorrhea of 71.1% [1]. Dysmenorrhea interferes with daily activities, including school or work absences and reduced productivity [1,2], and negatively affects the quality of life [3]. Medications, such as analgesics, are typically the first-line treatment [4,5], but they are often ineffective for many women [6]. Alternative treatments, such as heat application and lifestyle modifications, have also been suggested as options for managing dysmenorrhea [4,7].

Identifying risk factors for dysmenorrhea and positively addressing modifiable factors have been proposed as key strategies for managing the condition [5,7]. Known risk factors include biological factors (e.g., family history of dysmenorrhea, early menarche, menstrual volume), lifestyle (e.g., smoking, alcohol consumption, caffeine intake, physical activity), stress, and poor sleep quality [7,8,9,10,11]. Among these, physical activity and sedentary behavior are modifiable lifestyle behaviors that may be targeted to manage dysmenorrhea.

Physical inactivity (i.e., insufficient moderate-to-vigorous physical activity or lack of exercise altogether) and sedentary behavior are distinct behaviors [10,12,13]. In the past decade, the association between sedentary behavior (i.e., prolonged sedentary time) and health outcomes has received increased attention. Prolonged sedentary time has been shown to elevate the risk of chronic diseases and all-cause mortality, independent of physical activity levels [12,13,14,15].

The pathophysiology of primary dysmenorrhea is characterized by increased prostaglandin production during the menstrual cycle, which leads to myometrial contractions, vasoconstriction, and inflammation [7]. These changes result in uterine ischemia, reduced blood flow, and pain hypersensitivity, contributing to menstrual pain [5,16].

Sedentary behavior may exacerbate dysmenorrhea by influencing its underlying pathophysiology. It has been reported that prolonged sitting can decrease blood flow in the lower limb arteries and increase inflammation [17,18]. Additionally, sedentary behavior may negatively impact other known risk factors for dysmenorrhea, such as poor sleep quality [12,17] and mental stress [19]. Taken together, these findings suggest that sedentary behavior may exacerbate dysmenorrhea, and reducing sedentary time, regardless of physical activity levels, could be beneficial. However, the association between sedentary behavior and dysmenorrhea has rarely been studied.

An association between physical activities and dysmenorrhea has been suggested [10,20,21,22], with exercise interventions shown to reduce the levels of C-reactive protein, prostaglandin E_2_, and prostaglandin F_2_α, thereby alleviating uterine contraction and inflammation [23]. Exercise has been shown to relieve menstrual pain in women with dysmenorrhea [12,23,24], and moderate-to-vigorous physical activity has been found to reduce menstrual pain, with physical activity levels negatively associated with menstrual pain [21,22]. In contrast, physically inactive women are more likely to experience dysmenorrhea [10].

Previous studies on physical activity and dysmenorrhea have primarily assessed menstrual pain intensity to evaluate dysmenorrhea [10,21,22]. Many women with dysmenorrhea also experience a range of other menstrual symptoms, such as headaches, nausea, vomiting, diarrhea, fatigue, dizziness, irritability, and nervousness, all of which are linked to increased prostaglandin levels [7,25]. These symptoms, in addition to pain, can negatively impact daily activities [7,25]. Therefore, measuring pain alone may not fully capture the extent of dysmenorrhea. A multidimensional approach, including the assessment of both menstrual symptoms and pain, would likely provide a more comprehensive understanding of the condition [26]. Furthermore, there is a notable lack of studies on the association between sedentary behavior and these broader menstrual symptoms.

The aim of this study was to investigate whether sedentary behavior is independently associated with primary dysmenorrhea, as assessed by menstrual pain and the frequency and severity of menstrual symptoms, while controlling for moderate-to-vigorous physical activity levels and other potential risk factors in young Korean women.

## 2. Materials and Methods

### 2.1. Study Design

Ethical approval for this study was obtained from the Institutional Review Board of Kyonggi University (KGU-20210527-HR-069-02). Data were collected online from April to October 2023, and a survey was administered using the Google Forms platform. The survey was accessible via desktop, tablet, and mobile devices, ensuring participants from different technological backgrounds could easily participate. Participants received a Starbucks coffee coupon as an incentive.

Participants were recruited using convenience sampling via social media platforms and offline methods. The online survey was announced in the recruitment notice, which had been approved by the IRB; on social media platforms, such as young women’s communities on KakaoTalk, Naver Café, and Daum Café; and blogs featuring various businesses (such as esthetic clinics, gyms, etc.) run by the researcher’s acquaintances. Additionally, flyers were distributed at a large church and through the University Women’s Student Association at Kyonggi University and Myongji University, all located in Seoul and Gyeonggi Province.

The survey was distributed via a linked URL and QR code. Participants who wished to join were instructed to access the survey through the URL address or QR code in the recruitment notice. Prior to completing the survey, all participants were required to read an informed consent form, and only those who checked ‘Agree’ regarding participation proceeded with the anonymous survey. After completing the survey, participants submitted their answers along with their phone numbers through a Google Form to receive a gifticon as compensation. The research coordinator then sent a coffee coupon as a reward to the participants’ mobile phones.

The online survey included questions on menstrual pain intensity, menstrual symptoms, sedentary behavior, physical activity, general characteristics, and known risk factors for dysmenorrhea. The average time to complete the survey was approximately 20 min. A pilot test was conducted with 10 participants to assess the clarity of the questions and the functionality of the survey platform. Based on their feedback, minor adjustments were made to the wording of the questions to improve understanding.

### 2.2. Study Participants

**Figure 1:**
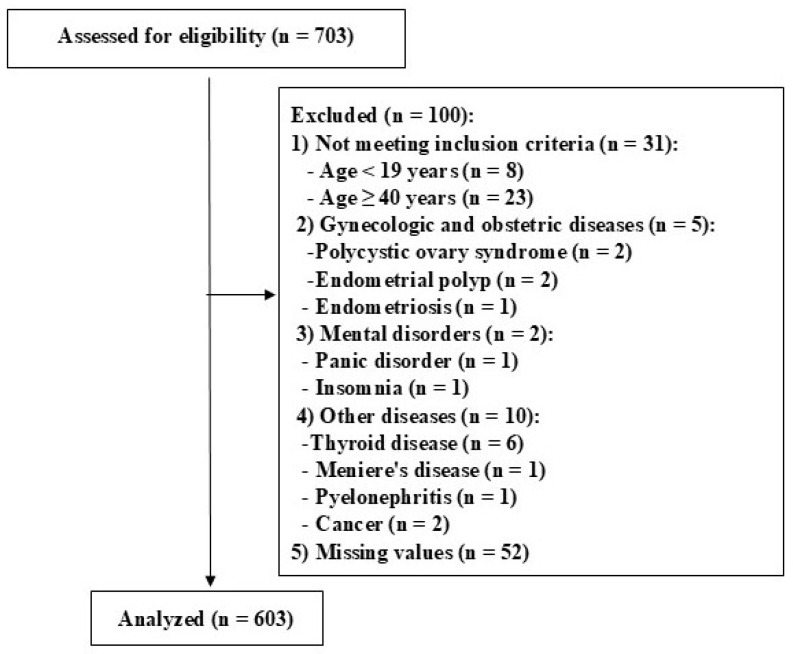
The study flowchart.

Women aged 19 to 39 years, with or without primary dysmenorrhea, were recruited for this study. A total of 703 women responded to the online survey, but data from 100 women were excluded from the analysis. The study flowchart and the reasons for exclusion are presented in Figure 1. Finally, 603 women were included in the data analysis.

### 2.3. General Characteristics and Dysmenorrhea Risk Factors

General subject characteristics included age, body mass index, education, occupation, marital status, monthly family income, perceived health status, existing diseases (multiple choice), medications (multiple choice), and chronic pain lasting more than 3 months (multiple choice). Known risk factors for dysmenorrhea included childbirth, a family history (i.e., sister or mother experiencing dysmenorrhea), early menarche before age 12, menstrual periods lasting more than 7 days, menstrual cycles longer than 35 days, irregular menstrual cycles, heavy menstrual flow, caffeine consumption, smoking, alcohol consumption, lack of physical activity, poor sleep quality, and stress, as identified in the literature [7,8,27].

Stress levels were assessed using the Korean version of the 10-item Perceived Stress Scale (PSS) [28,29]. A higher total PSS score (range 0–40) indicates greater stress [29]. Sleep quality was assessed using the Korean version of the Pittsburgh Sleep Quality Index (PSQI) [30,31]. A higher total PSQI score (range 0–21) indicates poorer sleep quality, with a score above 5 indicating poor sleep quality [30].

### 2.4. Menstrual Pain and Symptoms

Dysmenorrhea was assessed by measuring menstrual pain intensity and symptoms. Pain intensity was evaluated using a numeric rating scale (NRS), with menstrual pain categorized as ‘no pain’ (NRS = 0), mild (1–3), moderate (4–6), or severe (7–10) [10]. Menstrual symptoms were assessed using the Korean version of the Cox Menstrual Symptom Scale [32,33]. This scale evaluates the severity and frequency of 18 symptoms related to dysmenorrhea, including cramps, headaches, backaches, leg aches, abdominal pain, general aching, nausea, vomiting, dizziness, loss of appetite, diarrhea, facial blemishes, flushing, weakness, sleeplessness, depression, irritability, and nervousness [11,32]. Each symptom was rated for severity (not noticeable, slightly bothersome, moderately bothersome, severely bothersome, or very severely bothersome) and frequency (did not occur, lasted less than 3 h, lasted 3–7 h, lasted an entire day, or lasted several days) on a scale from 0 to 4 [11]. The total scores for severity and frequency were calculated separately.

### 2.5. Sedentary Behavior and Physical Activity

Sedentary behavior and physical activity were assessed using the Korean version of the Global Physical Activity Questionnaire (GPAQ) [34,35], which has been shown to be both reliable and valid [36,37]. The GPAQ assesses sedentary behavior with the following question: ‘The following question is about sitting or reclining at work, at home, during travel, or while socializing, including time spent sitting at a desk, with friends, in a car, bus, or train, reading, playing cards, or watching television—excluding time spent sleeping. How much time do you usually spend sitting or reclining on a typical day?’.

The GPAQ collects information on the days and time spent engaging in moderate and vigorous physical activity at work, during recreation, and for transportation (e.g., walking or cycling) in a typical week. Physical activity was converted to the metabolic equivalent of task (MET): 4 METs for moderate activities and transportation and 8 METs for vigorous activities, according to the GPAQ scoring protocol [34].

After calculating the MET-minutes per week for each activity in the domains of work, travel, and recreation, the MET-minutes from all three domains were summed to obtain the total MET-minutes per week. Weekly physical activity was categorized as ≥600 MET-minutes or <600 MET-minutes based on WHO recommendations for optimal health benefits, which combine moderate and vigorous physical activities to meet at least 600 MET-minutes per week [34].

### 2.6. Statistical Analysis

The data were analyzed using SPSS version 27.0 (IBM SPSS Statistics, Armonk, NY, USA). The significance level was set at 0.05 for all analyses. Independent *t*-tests for continuous variables and Chi-square tests for categorical variables were used to assess group differences in participants’ characteristics, dysmenorrhea risk factors, and menstrual pain and symptoms between the severe and less severe pain groups. Correlations among menstrual variables (NRS, severity, and frequency of menstrual symptoms), sedentary behavior, physical activity levels, PSQI total scores, and PSS scores were assessed using Pearson correlation coefficients. Stepwise linear regression analyses were conducted to identify covariates for the severity and frequency of menstrual symptoms, as well as menstrual pain intensity, based on general characteristics and known risk factors for dysmenorrhea. Additionally, forward Wald logistic regression analyses were performed to identify covariates associated with categorized pain intensity (less severe vs. severe pain).

Finally, multiple linear regression analyses were conducted to determine whether sedentary behavior independently is associated with the severity and frequency of menstrual symptoms, after controlling for weekly moderate-to-vigorous physical activity levels (<600 MET-minutes vs. ≥600 MET-minutes), and other covariates: family history of dysmenorrhea (no/unknown vs. yes), early menarche (<12 years vs. ≥12 years), menstrual cycle length (>35 days vs. ≤35 days), caffeine consumption (none vs. ≥1 drink), monthly family income (≥3.5 million vs. <3.5 million), chronic pain (no vs. yes), existing diseases (no vs. yes), body mass index, sleep quality (poor vs. good), and perceived stress scores. Multicollinearity among independent variables was assessed using tolerance values (cutoff > 0.1) and variance inflation factors (VIF < 10). No multicollinearity was found in any of the regression analyses. Additionally, multiple logistic regression was performed to assess whether sedentary behavior independently affects the severity of menstrual pain, adjusting for physical activity and other covariates. Adjusted odds ratios for severe pain were calculated with 95% confidence intervals.

## 3. Results

### 3.1. Participant Characteristics

The characteristics of 603 study participants are presented in Table 1. Most participants reported mild-to-moderate menstrual pain (66.2%) and severe pain (24.7%), while 9.1% reported no pain. There was no significant difference in participant characteristics between the no pain/mild pain group and the moderate pain group. Therefore, to facilitate comparison, participants were divided into two groups: the less severe pain group (no, mild, and moderate pain) and the severe pain group.

Among the general characteristics, the severe pain group had a higher body mass index (*p* < 0.001), lower monthly household income (*p* < 0.001), and a higher proportion of chronic pain (*p* < 0.01) compared to the less severe pain group. Among the menstrual and lifestyle characteristics, significant group differences were found in menarcheal age (*p* < 0.05), volume of menstrual fluid (*p* < 0.05), family history of dysmenorrhea (*p* < 0.001), intensity of menstrual pain (*p* < 0.001), severity (*p* < 0.001) and frequency (*p* < 0.001) of menstrual symptoms, alcohol consumption (*p* < 0.01), binge drinking (*p* < 0.001), caffeine consumption (*p* < 0.001), sleep quality (*p* < 0.001), stress (*p* < 0.001), and sedentary time (*p* < 0.05). These results reveal that the proportions of early menarche, heavy menstrual volume, family history of dysmenorrhea, alcohol consumption, binge drinking, caffeine consumption, and poor sleep quality were higher in the severe pain group. The severe pain group also had a higher stress level, longer sedentary time, and experienced more severe and frequent menstrual symptoms. There were no significant group differences in other general, menstrual, and lifestyle characteristics.

A total of 530 women (87.9%) reported having no existing diseases, while 73 women (12.1%) had one or more conditions, including heart disease (*n* = 3), dyslipidemia (*n* = 3), allergies (*n* = 27), diabetes mellitus (*n* = 5), hypertension (*n* = 7), arthritis (*n* = 15), cerebrovascular disease (*n* = 4), cancer (*n* = 1), chronic respiratory disease (*n* = 3), hypotension (*n* = 3), gastritis (*n* = 3), Crohn’s disease (*n* = 1), dermatosis (*n* = 1), and varicose veins (*n* = 1). Thirty-nine women (6.5%) were on medication. Additionally, 34% of women (*n* = 205) reported experiencing one or more types of chronic pain, including headache (*n* = 51), whole-body aches (*n* = 4), back pain (*n* = 57), neck pain (*n* = 51), shoulder pain (*n* = 78), wrist pain (*n* = 42), knee pain (*n* = 32), ankle pain (*n* = 19), foot pain (*n* = 10), and other pain (*n* = 6).

### 3.2. Correlations Among Sedentary Time, Menstrual Pain and Symptoms, and Covariates

The Pearson correlation coefficients are presented in Table 2. Longer sitting time was associated with more severe (*p* < 0.01) and frequent (*p* < 0.001) menstrual symptoms but not with menstrual pain intensity measured by the NRS. A higher level of moderate-to-vigorous physical activity (total MET-minutes per week) was associated with more frequent menstrual symptoms (*p* < 0.05); a higher PSQI total score, indicating worse sleep quality (*p* < 0.01); and shorter sedentary time (*p* < 0.001). Additionally, higher PSQI and PSS scores were correlated with more severe menstrual pain, as well as more severe and frequent menstrual symptoms (all *p* < 0.05 or *p* < 0.001).

### 3.3. Associations Between Covariates and Menstrual Variables

Factors associated with menstrual variables, among general characteristics and known risk factors for dysmenorrhea, were included as covariates: body mass index, monthly family income, chronic pain, existing diseases, family history of dysmenorrhea, early menarche, menstrual cycle, volume of menstrual fluid, caffeine consumption, sleep quality, and stress. Stepwise linear regression analyses revealed that a family monthly income of less than 3.5 million, chronic pain, a family history of dysmenorrhea, a menstrual cycle of 35 days or fewer, caffeine consumption, poor sleep quality, and higher stress were all associated with an increased frequency and severity of menstrual symptoms (all *p* < 0.01 or *p* < 0.001). Additionally, a higher body mass index was associated with an increased frequency of symptoms (*p* < 0.01), while existing diseases were associated with increased severity (*p* < 0.05).

Higher menstrual pain intensity was associated with chronic pain (*p* < 0.001), a family history of dysmenorrhea (*p* < 0.001), early menarche before 12 years of age (*p* < 0.05), caffeine consumption (*p* < 0.01), and poor sleep quality (*p* < 0.001). Multiple logistic regression analysis revealed that a family monthly income of less than 3.5 million (*p* < 0.01), a family history of dysmenorrhea (*p* < 0.001), early menarche before 12 years (*p* < 0.05), caffeine consumption (*p* < 0.001), and poor sleep quality (*p* < 0.001) increased the risk of menstrual pain becoming severe.

### 3.4. Association Between Sedentary Time and Menstrual Variables

The results of unstandardized and standardized regression coefficients from multiple regression analyses for the predictors of menstrual pain intensity and the severity and frequency of menstrual symptoms are presented in Table 3. Multiple linear regression analyses showed that sedentary time was associated with the severity (*p* < 0.05) and frequency (*p* < 0.01) of menstrual symptoms but not with menstrual pain intensity after controlling for moderate-to-vigorous physical activity and other covariates. However, multiple logistic regression analysis revealed that sedentary time increased the adjusted odds ratio for severe menstrual pain compared to less severe pain (*p* < 0.05), after controlling for moderate-to-vigorous and other covariates. These findings indicate that women with longer sedentary time are more likely to experience more severe and frequent menstrual symptoms, as well as more severe menstrual pain.

## 4. Discussion

This study investigated the associations between sedentary behavior and various aspects of primary dysmenorrhea, including menstrual pain intensity, the likelihood of experiencing severe pain, and the frequency and severity of menstrual symptoms, in young Korean women aged 19 to 39 years. The findings indicated that longer periods of sedentary behavior were associated with more severe menstrual pain, as well as an increased frequency and severity of menstrual symptoms, independent of moderate-to-vigorous physical activity levels and other known risk factors for dysmenorrhea.

Sedentary behavior may exacerbate primary dysmenorrhea by influencing its underlying mechanisms, such as uterine ischemia resulting from increased levels of prostaglandin F2α and prostaglandin E2, reduced blood flow, inflammation, and heightened pain sensitivity, ultimately leading to increased pain [5,16]. While no studies have specifically examined the impact of sedentary behavior on the pathophysiology of dysmenorrhea, it has been suggested that sedentary behavior negatively affects nociception and pain modulation in individuals with chronic pain conditions, such as low back pain and fibromyalgia [38,39,40]. Chronic pain is known to be associated with hypersensitivity to painful stimuli and reduced endogenous pain inhibition [39], a phenomenon similar to the pain hypersensitivity that persists throughout the menstrual cycle in women with dysmenorrhea, suggesting a dysfunctional endogenous analgesic system [41]. Sedentary behavior has been shown to worsen chronic low back pain [38] and negatively impact brain regions involved in pain regulation, including those responsible for pain processing and modulation, in women with fibromyalgia [40]. Additionally, sedentary behavior has been linked to decreased blood flow in the arteries of the lower limbs and increased inflammation in healthy adults [17,18]. Therefore, sedentary behavior could potentially exacerbate dysmenorrhea by amplifying pain hypersensitivity, reducing the descending inhibition of nociception, and increasing uterine ischemia.

Adults spend approximately 8 h per day in sedentary behavior [42,43]. While sedentary behavior is associated with increased health risks, no specific or quantified threshold for sedentary time has been established, nor are there clear recommendations on how to break up sedentary periods [42]. Recently, Hartman et al. [44] reported that reducing sedentary time by just one hour—from 10 h to 9 h—results in significant increases in both peripheral and cerebral blood flow. A recent scoping review [45] also found that prolonged sedentary behavior leads to reduced blood flow in the lower limbs. Interestingly, low-intensity physical activity—rather than high-intensity activity—appears to be more effective in mitigating vascular dysfunction in the legs [45]. In our study, we found that women with severe menstrual pain averaged 9 h of sedentary time, compared to 8 h for those with less severe pain. Therefore, it is plausible that reducing sedentary time or incorporating breaks with even low-intensity physical activity could help alleviate menstrual pain.

Since prostaglandins are present throughout the body, elevated levels during menstruation often lead to systemic symptoms, such as nausea, vomiting, and diarrhea, which commonly accompany menstrual pain in women with dysmenorrhea [7,25,46]. While no studies have specifically investigated the association between sedentary behavior and these menstrual symptoms, our findings indicate that sedentary behavior is negatively associated with both the frequency and severity of menstrual symptoms, even after controlling for moderate-to-vigorous physical activity levels and other known risk factors for dysmenorrhea. No specific cutoff score has been established for classifying the frequency and severity of menstrual symptoms. Correlation analyses also revealed that longer sedentary time was associated with more frequent and severe menstrual symptoms.

In this study, moderate-to-vigorous physical activity showed no significant association with menstrual pain or symptoms, whereas sedentary behavior did. Although exercise interventions have been shown to be effective in alleviating dysmenorrhea [20], the results of cross-sectional studies examining the association between physical activity and dysmenorrhea remain inconclusive. Some studies suggest a potential link between high levels of physical activity and reduced menstrual pain [10,21,47], while others report no significant relationship [48,49]. This inconsistency may be attributed to the cross-sectional design of these studies, including the present one. Further longitudinal research, such as prospective cohort studies, is needed to better understand this relationship. Another possible explanation may lie in the nature and measurement of each variable. Physical activity levels can vary in type, intensity, and frequency, whereas sedentary behavior, which is typically characterized by sitting time, is more consistently defined and measured. As a result, the inverse association between sedentary behavior and menstrual symptoms may be more detectable in cross-sectional data.

We initially hypothesized that sedentary behavior might also be associated with sleep quality and mental stress, both of which are known risk factors for dysmenorrhea [12,17,19]. However, the correlation analyses showed no significant association between sedentary time and either sleep quality or stress scores. In contrast, poorer sleep quality and higher stress levels were significantly associated with more frequent and severe menstrual symptoms, as well as more intense menstrual pain. In our study, poor sleep quality emerged as a strong risk factor for both menstrual symptoms and pain, even after adjusting for covariates. Additionally, stress was found to be a risk factor for menstrual symptoms but not for the intensity of menstrual pain, after controlling for other dysmenorrhea risk factors. Consistent with our results, poor sleep quality and high stress have previously been shown to impact dysmenorrhea [8,11].

Questionnaires and objective tools, such as accelerometers and pedometers, are widely used to assess sedentary behavior and physical activity [50]. Among these, the GPAQ, developed by the World Health Organization, is one of the most commonly used tools [50]. It measures moderate-to-vigorous physical activity across work, transportation, and recreational activities, as well as sedentary time [51]. However, one limitation of the GPAQ is the potential for recall bias in self-reported measurements. Previous studies have also found that accelerometer-based measurements of sedentary behavior tend to be significantly higher than those reported by participants using the GPAQ [51,52]. Thus, sedentary time may have been underestimated in our study compared to accelerometer data. For this reason, incorporating accelerometers in future studies is recommended.

Oral contraceptives are used to treat dysmenorrhea, and their use has been suggested to alleviate dysmenorrhea [7,27]. However, we did not assess whether participants were using contraceptives, which may represent a limitation, as it could have influenced the severity of dysmenorrhea. Another limitation is the use of convenience sampling, which may limit the generalizability of the findings. Additionally, although the survey was designed to be accessible across devices, participants with limited internet access may have been excluded from this study.

Nonetheless, this study has a key strength: it demonstrates that sedentary time is independently associated with dysmenorrhea, even after accounting for the potential confounding effects of moderate-to-vigorous physical activity (including work, commuting, and recreation) and other known risk factors. It provides the first insights into how sedentary time is associated with both the frequency and severity of menstrual symptoms, as well as menstrual pain.

Longitudinal studies, particularly those investigating the impact of interventions to reduce sedentary behavior, along with cohort studies, are needed to establish the causal relationship between sedentary behavior and dysmenorrhea. Furthermore, the effects of sedentary behavior on prostaglandins, inflammation, and hormones—factors related to the pathophysiology of dysmenorrhea [16]—should be examined to better understand the underlying mechanisms.

## 5. Conclusions

This study highlights the importance of reducing sedentary time in young women to manage dysmenorrhea. To optimize physical activity in women with dysmenorrhea, it is recommended to decrease sedentary time and incorporate regular breaks, regardless of an increase in physical activity levels.

## Figures and Tables

**Table 1:** Participant characteristics.

Variables	Categories	Less Severe Pain (*n* = 454)	Severe Pain (*n* = 149)	*p*
Intensity of menstrual pain		3.38 ± 2.0	7.72 ± 0.9	0.000 ^***^
Severity of menstrual symptoms		6.22 ± 7.6	17.59 ± 11.4	0.000 ^***^
Frequency of menstrual symptoms		7.18 ± 8.7	21.18 ± 12.6	0.000 ^***^
Sedentary time (hours/day)		8.07 ± 3.9	9.06 ± 4.9	0.013 ^*^
Total MVPA (MET-minutes/week)		3077.93 ± 4532.2	2995.97 ± 4189.9	0.845
Weekly MVPA	<600 MET-minutes	92 (20.3)	40 (26.8)	0.092
	≥600 MET-minutes	362 (79.7)	109 (73.2)	
Age (years)		28.16 ± 5.5	28.14 ± 5.5	0.601
Body mass index (kg/m^2^)		20.55 ± 3.3	21.81 ± 3.4	0.000 ^***^
Education	High school graduate	133 (29.3)	45 (30.2)	0.833
	College or higher	321 (70.7)	104 (69.8)	
Job	Unemployed	162 (35.7)	54 (36.2)	0.902
	Employed	292 (64.3)	95 (63.8)	
Monthly family income (KRW)	<2.5 million	53 (11.7)	29 (19.5)	0.000 ^***^
	2.5~3.5 million	42 (9.3)	31 (20.8)	
	3.5~5.5 million	158 (34.8)	41 (27.5)	
	≥5.5 million	201 (44.3)	48 (32.2)	
Marital status	Married/others	112 (24.7)	35 (23.5)	0.771
	Unmarried	342 (75.3)	114 (76.5)	
Childbirth	No	365 (80.4)	124 (83.2)	0.445
	Yes	89 (19.6)	25 (16.8)	
Existing disease	No	399 (87.9)	131 (87.9)	0.991
	Yes	55 (12.1)	18 (12.1)	
Medication	No	426 (93.8)	138 (92.6)	0.601
	Yes	28 (6.2)	11 (7.4)	
Chronic pain	No	317 (69.8)	81 (54.4)	0.001 ^**^
	Yes	137 (30.2)	68 (45.6)	
Early menarche	Yes (<12 years)	88 (19.4)	41 (27.5)	0.036 ^*^
	No (≥12 years)	366 (80.6)	108 (72.5)	
Period regularity	Regular	300 (66.1)	103 (69.1)	0.493
	Irregular	154 (33.9)	46 (30.9)	
Menstrual cycle	≤20 days	9 (2.0)	4 (2.7)	0.080
	21–35 days	339 (74.7)	123 (82.6)	
	>35 days	106 (23.3)	22 (14.8)	
Menstrual fluid	Light	91 (20.0)	17 (11.4)	0.016 ^*^
	Moderate	263 (57.9)	86 (57.7)	
	Heavy	100 (22.0)	46 (30.9)	
Period length	<7 days	445 (98.0)	144 (96.6)	0.334
	>7 days	9 (2.0)	5 (3.4)	
Family history of dysmenorrhea	No	83 (18.3)	20 (13.4)	0.000 ^***^
	Yes	235 (51.8)	114 (76.5)	
	Unknown	136 (30.0)	15 (10.1)	
Smoking	No	369 (81.3)	128 (85.9)	0.198
	Yes	85 (18.7)	21 (14.1)	
Drinking	None	132 (29.1)	23 (15.4)	0.001 ^**^
	Yes	322 (70.9)	126 (84.6)	
Caffeine consumption	None	92 (20.3)	6 (4.0)	0.000 ^***^
	1–6 times/week	227 (50.0)	72 (48.3)	
	everyday	135 (29.7)	71 (47.7)	
PSQI total scores		4.6 ± 2.8	6.9 ± 3.7	0.000 ^***^
Sleep quality	Poor	142 (31.3)	93 (62.4)	0.000 ^***^
	Good	312 (68.7)	56 (37.6)	
Stress		15.95 ± 6.4	18.1 ± 5.8	0.000 ^***^

Data are presented as *n* (%) or mean ± standard deviation. ^*^ *p* < 0.05, ^**^ *p* < 0.01, ^***^ *p* < 0.001. Abbreviations: MVPA, moderate-to-vigorous physical activity; PSQI, Pittsburgh Sleep Quality Index.

**Table 2:** Pearson correlation coefficients (*r*) among sedentary time, menstrual variables, and covariates.

Variables	1	2	3	4	5	6	7
1. Intensity of menstrual pain	1						
2. Severity of menstrual symptom	0.484 ^***^	1					
3. Frequency of menstrual symptom	0.519 ^***^	0.877 ^***^	1				
4. Sedentary time (hours/day)	0.059	0.123 ^**^	0.144 ^***^	1			
5. Total MVPA (MET-minutes/week)	0.010	0.067	0.096 ^*^	−0.237 ^***^	1		
6. Sleep quality	0.260 ^***^	0.496 ^***^	0.509 ^***^	0.067	0.140 ^**^	1	
7. Stress	0.095 ^*^	0.284 ^***^	0.264 ^***^	0.069	0.044	0.324 ^***^	1

^*^ *p* < 0.05, ^**^ *p* < 0.01, ^***^ *p* < 0.001. *n* = 603. Abbreviations: MVPA, moderate-to-vigorous physical activity.

**Table 3:** Associations between sedentary time and menstrual variables.

Dependent Variables	Intensity of Menstrual Pain		Severity of Menstrual Symptom		Frequency of Menstrual Symptom		Less Severe Pain ^b^ vs. Severe Pain
Independent Variables	*B* (SE)	*β*	*B* (SE)	*β*	*B* (SE)	*β*	Adjusted OR (95% CI)
Sedentary time (hours/day)	0.02 (0.02)	0.04	0.19 (0.08)	0.08 ^*^	0.29 (0.09)	0.10 ^**^	1.05 (1.00–1.10) ^*^
Weekly MVPA ^a^	0.10 (0.25)	0.02	−0.32 (0.84)	−0.01	0.09 (0.96)	0.00	1.05 (0.64–1.75)
Body mass index (kg/m^2^)	0.04 (0.03)	0.05	0.18 (0.11)	0.06	0.32 (0.12)	0.09 ^*^	1.03 (0.96–1.09)
Monthly family income ^a^	−0.29 (0.23)	−0.05	−2.48 (0.81)	−0.11 ^**^	−2.92 (0.92)	−0.11 ^**^	1.87 (1.18–2.96) ^**^
Existing disease ^a^	−0.26 (0.31)	−0.03	2.33 (1.06)	0.08 ^*^	1.85 (1.21)	0.05	0.54 (0.27–1.05)
Chronic pain ^a^	0.72 (0.21)	0.13 ^**^	3.79 (0.73)	0.18 ^***^	4.48 (0.84)	0.18 ^***^	1.51 (0.98–2.34)
Early menarche ^a^	−0.63 (0.24)	−0.10 ^*^	−0.42 (0.82)	−0.02	−1.63 (0.93)	−0.06	1.83 (1.12–2.97) ^*^
Menstrual cycle ^a^	−0.36 (0.24)	−0.07	−3.39 (0.83)	−0.14 ^***^	−3.15 (0.94)	−0.11 ^**^	1.60 (0.91–2.80)
Family history of dysmenorrhea ^a^	1.15 (0.20)	0.22 ^***^	2.13 (0.68)	0.11 ^**^	3.25 (0.78)	0.14 ^***^	3.12 (1.97–4.94) ^***^
Caffeine consumption ^a^	0.73 (0.27)	0.10 ^**^	2.64 (0.92)	0.10 ^**^	3.04 (1.05)	0.10 ^**^	5.05 (2.08–12.24) ^***^
Sleep quality ^a^	0.92 (0.22)	0.18 ^***^	5.93 (0.75)	0.29 ^***^	6.99 (0.85)	0.30 ^***^	2.75 (1.77–4.30) ^***^
Stress	0.00 (0.02)	0.00	0.21 (0.06)	0.13 ^***^	0.20 (0.06)	0.11 ^**^	1.02 (0.99–1.06)
	*F* (12, 590) = 9.94, Adjusted *R*^2^ = 0.151, *p* < 0.001		*F* (12, 590) = 25.75, Adjusted *R*^2^ = 0.330, *p* < 0.001		*F* (12, 590) = 27.25, Adjusted *R*^2^ = 0.343, *p* < 0.001		Nagelkerke pseudo *R*^2^ = 0.277, *p* < 0.001

^a^**^.^** Dummy variables. ^b^**^.^** Hosmer and Lemeshow test: *p* > 0.05. Abbreviations: *B*, unstandardized regression coefficient; SE, standard error; *β*, standardized regression coefficient; OR, odds ratio; CI, confidence interval; MVPA, moderate-to-vigorous physical activity. ^*^ *p* < 0.05, ^**^ *p* < 0.01, ^***^ *p* < 0.001. *P* values less than 0.05 are in bold. *n* = 603.

## Data Availability

The data presented in this study are available on request from the corresponding author because the data are part of an ongoing study and are not yet publicly available.

## Abbreviations

The following abbreviations are used in this manuscript:

GPAQ	Global Physical Activity Questionnaire
NRS	Numeric rating scale
MET	Metabolic equivalent of task
PSQI	Pittsburgh Sleep Quality Index
PSS	Perceived Stress Scale
